# Supplementary material for: Plantar flexor muscle size, composition and strength and the redistribution of joint work in gait of healthy older adults
Source: PeerJ. 2026 Sep 24;14:e21734. doi: 10.7717/peerj.21734 (PMC13616193; doi:10.7717/peerj.21734)
Supplement: Supplementary file 1 [file peerj-14-21734-s003.docx]

Supplement A: Detailed list of the study inclusion and exclusion criteria for younger and older adult participants. Participants completed a telephone screening interview to evaluate eligibility for the study.

Inclusion Criteria:

- Generally healthy, by self-report
- Ambulatory without the use of walking aids
- Living independently in the community
- Non-smokers (nicotine and marijuana), for at least the preceding 2 years

Exclusion Criteria:

- Pregnancy, or <12 months post-partum, determined by self-report
- History of major neurological or neuromuscular condition that may impact physical function, including cerebrovascular disease, peripheral neuropathy, neurodegenerative disease, demyelinating disease, cerebellar or extrapyramidal disease, etc.
- History of myocardial infarction, angina, peripheral vascular disease, surgical or percutaneous coronary artery revascularization or severe pulmonary disease.
- History of rheumatoid arthritis
- History of diabetes or other metabolic disease that may impact neuromuscular function • Uncontrolled hypertension (blood pressure >179/109)
- History of anterior cruciate ligament injury
- Moderate to severe lower extremity osteoarthritis or pain with Knee or Hip Injury Osteoarthritis Outcome Score- Pain subscale (KOOS;HOOS) of <75. (i.e., pain on level walking or that limits activities of daily living such as household ambulation and self-care).
- Short physical performance battery score (SPPB) <7
- Symptoms of pain, muscle cramps, joint stiffness, dyspnea, angina, light-headedness or other contraindications to exercise participation.
- The use of beta-blockers, sedatives, tranquilizers, or other medication that may impair physical function
- The use of statin medications that result in symptoms of muscle pain or myopathy
- Body-mass index <18 kg/m2 or >35 kg/m2, may suggest an underlying metabolic problem and increased fat mass may alter mobility function
- Unintentional weight loss of greater than 2.5 kg during the last 3 months
- The use of hormone replacement therapy in older participants, as this treatment is an attempt to circumvent normal age-related declines in sex hormone levels and may affect our measures
- Participation in a weight loss or exercise training program in the last year
- An inability to understand written and spoken English
- An inability to follow instructions, as determined by the investigators during the consenting process
- Moderate to severe COVID-19 infection requiring medical intervention or hospitalization • Residual COVID-19 symptoms such as breathing trouble, fogginess and fatigue
- History of substance abuse requiring medical/pharmacological support.
